# 1230. Optimization of a Public-Facing Health System Antimicrobial Stewardship Website

**DOI:** 10.1093/ofid/ofad500.1070

**Published:** 2023-11-27

**Authors:** Christie M Bertram, Sheila K Wang, William J Moore, Jaime Borkowski, Erin Weslander

**Affiliations:** Northwestern Memorial Hospital/Rosalind Franklin University of Medicine and Science, Chicago, Illinois; Midwestern University College of Pharmacy/Northwestern Memorial Hosptial, Downers Grove, Illinois; Northwestern Medicine, Chicago, Illinois; NM Delnor Hospital, Geneva, Illinois; Northwestern Memorial Hospital, Chicago, Illinois

## Abstract

**Background:**

Following health system integration of an academic medical center and 10 community hospitals, efforts were made to unify the individual antimicrobial stewardship programs (ASP) and standardize resources within a system-wide Antimicrobial and Diagnostic Stewardship Program (NM ADSP). We assessed the impact of optimizing our public-facing website with updated resources, improving platform usability and convenience, and promoting the new ADSP website to stakeholders.

**Methods:**

Previously conducted focus group sessions of the general public and internal questionnaires to clinicians were used to assess the value of antimicrobial stewardship (AS) and available resources/education. Our system ADSP team worked to assess and merge existing AS resources across each site. Concurrently, the ADSP team partnered with marketing specialists and digital content creators at our institution to produce and launch the new ADSP website. Tracking of total website pageviews per week for 3 months before and 3 months after the ADSP website optimization was evaluated.

**Results:**

Feedback from focus groups and clinical users confirmed the value of a website as a convenient and accessible platform in disseminating important AS resources and education to a broad audience. From these assessments, we identified and prioritized high-use AS resources and education content across the system (e.g. allergy, antibiograms, antimicrobial dosing, COVID-19, and guidelines/protocols) and prioritized these on the home page. Other information identified as useful included site-specific practice guidance, about us, clinical & patient education, news/events, and residency/fellowship information, which were also posted on the home page. A significant difference in the median unique website views per week was observed after optimization and launch of the new ADSP website (pre: 347; post: 524, *p* < 0.001).

NM ADSP Website Resources

**Figure:**
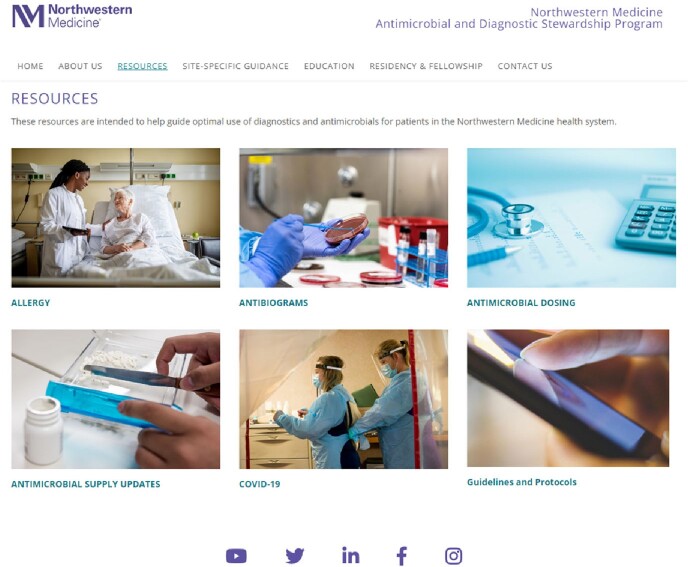

**Conclusion:**

Antimicrobial stewardship is a collective effort. Establishing an organized and resourceful AS public-facing website that is user-friendly and reliable for our stakeholders betters our chances of improving the use of anti-infectives and reducing the threat of antimicrobial resistance. Our experience may be useful to identify educational best practices for enhanced ASP impact at other institutions.

**Disclosures:**

**All Authors**: No reported disclosures

